# Image Findings from Dual-phase Computed Tomography Pulmonary Angiography for Diagnosing Tuberculosis-associated Fibrosing Mediastinitis

**DOI:** 10.2174/0115734056324457241218113104

**Published:** 2025-01-02

**Authors:** Mengdi Zhang, Chao Bu, Kaiyu Jiang, Xiaozhou Long, Zhonghua Sun, Yunshan Cao, Yu Li

**Affiliations:** 1 Department of Radiology, The Seventh Affiliated Hospital of Sun Yat-sen University, 628 Zhenyuan Rd., Guangming District, Shenzhen 518107, China; 2 Department of Cardiology, Pulmonary Vascular Disease Center, Gansu Provincial Hospital, Lanzhou 730000, China; 3 Curtin Medical School, Curtin University, Perth 6102, Western Australia, Australia; 4 Curtin Medical Research Institute (Curtin MRI), Curtin University, Perth 6102, Western Australia, Australia; 5 Heart, Lung and Vessels Center, Sichuan Provincial People's Hospital, University of Electronic Science and Technology of China, 610072, China

**Keywords:** Computed tomography, Fibrosing mediastinitis, Diagnosis, Tuberculosis, Imaging, Angiography

## Abstract

**Objective:**

Fibrosing mediastinitis (FM) is a rare and benign disease affecting the mediastinum and often causes pulmonary hypertension (PH). Timely diagnosis of PH caused by FM is clinically important to mitigate complications such as right heart failure in affected individuals. This retrospective study aimed to analyze the CT imaging characteristics of tuberculosis (TB) related FM in patients with (TB). Additionally, the study investigates the underlying reasons contributing to the manifestation of symptoms.

**Methods:**

From April 2007 to October 2020, high-resolution CT (HRCT) and dual-phase CT pulmonary angiography images of 64 patients with suspected FM diagnosed with PH at a tertiary hospital were examined. The imaging characteristics of these CT scans were analyzed, with a specific focus on the TB-FM involvement of the pulmonary veins, pulmonary arteries, and bronchi (down to the segment level).

**Results:**

HRCT imaging revealed that fibrous tissue inside the mediastinum exhibited minimal or negligible reinforcement in TB-FM and diffuse fibrous infiltration in the mediastinum and hilar areas. Notably, segmental bronchial and pulmonary artery stenosis are more pronounced and frequently co-occurring than lobe-level stenosis. Pulmonary venous stenosis developed outside the pericardium, whereas pulmonary artery stenosis occurred outside the mediastinal pleura. Furthermore, no isolated FM involvement in pulmonary veins was noticed in this cohort.

**Conclusion:**

HRCT imaging of TB-related FM presents unique features in certain regions of the bronchi, pulmonary veins, and pulmonary arteries. Thus, it is imperative to accurately identify fibrous tissue involvement in mediastinal lesions for proper diagnosis and management of TB-FM.

## 1 INTRODUCTION

Fibrosing mediastinitis (FM) is a rare and progressive condition characterized by a gradual wrapping and infiltration of mediastinal structures by benign proliferative fibrous tissues. This condition often leads to compression and occlusion of the tracheobronchial tree, pulmonary vessels, esophagus, and pericardium, consequently manifesting in complications such as atelectasis, obstructive pneumonia, pulmonary hypertension (PH), and even right heart failure among afflicted patients. Furthermore, if left untreated, PH, a serious and progressive hemodynamic disease, can result in a high fatality rate [1].

In China, tuberculosis (TB) stands as a primary cause of FM[(2]. The treatment of FM poses significant challenges as it includes medication, surgery, and endovascular interventions. Owing to the various etiologies and complex pathology associated with FM, the efficacy of drugs is limited, and surgical intervention carries a mortality rate as high as 20% [3, 4]. Meanwhile, endovascular interventions are now being used to ease symptoms caused by pulmonary vessel obstruction, which is the preferred treatment for FM [5-8]. Studies indicate that patients with FM may experience more complications from endovascular interventions compared to those with other diseases, resulting in pulmonary vascular stenosis. Moreover, it is suggested that the dense, often calcified tissue invading the vessel wall is the main cause of these complications [5]. Hence, the decision to perform balloon angioplasty or implant stents in patients with FM should only be made following a careful pre-procedure assessment. In this regard, contrast-enhanced CT and CT pulmonary angiography (CTPA) are the methods of choice for evaluating the involvement and severity of FM, as these can localize lesions in specific broncho-vascular bundles, aiding in the selection of an appropriate stent to cover the obstructed vessels.

In the past decade, the majority of studies on FM imaging modalities have concentrated on diagnosis, comprehensive imaging of the characteristics of obstructed structures, and establishing correlations between CT findings and clinical symptoms [9-17]. However, these studies have not revealed the imaging features of FM and the key features used to differentiate the diagnosis of CTPA. The reasons behind some features (atelectasis and pleural effusion) are also unclear. Thus, our study aims to provide a comprehensive imaging analysis and the extent of stenosis of affected structures in patients with FM. We seek to determine the diagnostic value of CTPA in TB-FM and to improve radiologists’ diagnostic accuracy for FM. Further, we hope to utilize these imaging characteristics to assist proceduralists in making informed decisions regarding surgical approaches and perioperative treatment to avoid potential complications.

## 2 MATERIALS AND METHODS

### 2.1 Study Sample

**Fig. (1):**
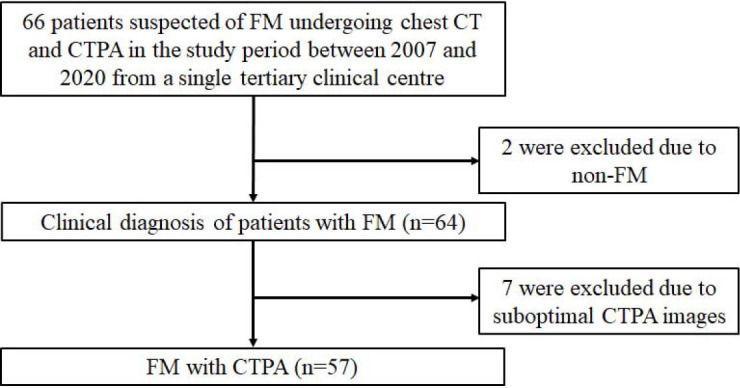
The study flowchart shows data collection for patients with FM. (FM= fibrosis mediastinitis).

From April 2007 to October 2020, we collected data from patients who were diagnosed with FM and treated at Gansu Provincial Hospital in Lanzhou, China. Inclusion criteria include patients with evidence of present or prior Mycobacterium tuberculosis infection, undergoing HRCT and CTPA, and a diagnosis of fibrosing mediastinitis on contrast-enhanced CT. Patients with a history of sarcoidosis or autoimmune diseases and those who underwent mediastinal surgery, radiation therapy, or were diagnosed with a mediastinal tumor were excluded. Examinations of *Histo-plasmacpsulatum* infection were not taken into account since China was not in the epidemic areas of histoplasmosis. Two patients who were diagnosed with non-FM were excluded from the study. Additionally, seven cases were removed from the vascular correlation analysis due to suboptimal CTPA imaging quality. Notably, the remaining 64 patients had no history of mediastinal radiation, mediastinal cancer, or autoimmune illness. According to the Chinese standard for pulmonary TB imaging diagnosis [18, 19], typical findings on lung HRCT included lymphadenopathy with or without calcification, pleural effusion, consolidation, cavitation, centrilobular and tree-in-bud nodules, miliary nodules, fibronodular scarring, peribronchial fibrosis, and bronchiectasis. If three of these symptoms are identified in a single patient alongside a clinical history, an imaging diagnosis of TB can be established. As a result, 64 individuals were diagnosed with TB based on HRCT findings (Fig. **1**). Among them, 41 cases of TB were confirmed using acid-fast bacilli staining, biopsy tissue examination, or sputum mycobacterial culture. Furthermore, only one patient developed an esophageal fistula after esophagectomy.

The institutional ethical review board approved the research protocol. As this study was retrospective, informed consent was not deemed necessary.

### 2.2 Clinical Data

Age, sex, symptoms, etiological variables, smoking history, WHO functional class, 6-min walk test, blood levels of C-reactive protein, and NT-proBNP were all considered as clinical data. The right atrial area (RAA), right ventricular area, and systolic pulmonary artery pressure (SPAP) were all measured using echocardiography (RVA). Meanwhile, a right heart catheter (RHC) examination was utilized to assess mixed venous saturation (SvO2), right atrial pressure (RAP), mean pulmonary arterial pressure (mPAP), pulmonary artery wedge pressure (PAwP), and pulmonary vascular resistance (PVR). A PAwP value less than 15 mmHg indicated precapillary PH, while a value exceeding 15 mmHg indicated postcapillary PH.

### 2.3 CT Scanning Protocols and Imaging Analysis

All CTPA scans were performed using a 192-slice dual-source CT scanner (Siemens Force, Siemens Healthcare, Forchheim, Germany). Standard acquisition parameters were employed: 104 mAs with automatic tube current modulation, a pitch of 1.7, rotating time of 0.25 s, reconstruction layer thickness of 1 mm, and interval of 0.7 mm. The intelligent tracking method was used to initiate the scanning, with the monitoring level set at the right atrium and a threshold of 60 HU. After reaching it, the first pulmonary artery phase was scanned within 2 s, followed by the second aortic phase, which was performed 5 s later. A total of 50−60 mL of contrast agent was administered at a rate of 4.5 mL/s, with normal saline injected at the same speed following administration of the contrast agent.

Three experienced radiologists (C.B., M.Z., and Y.L.), each with over five years of experience in interpreting chest CT images, assessed all chest HRCT and CTPA images. Multiplanar reformation (MPR) was utilized to examine the narrowing of arteries, veins, or bronchi, and grading of these images was conducted visually by these three observers.

The radiologists evaluated the main, right, and left pulmonary arteries, assessing the degree of stenosis in both inferior and superior pulmonary veins on the left and right sides. The most stenosed portion of the vasculature was preserved, and the assessment extended to the segmental and lobar pulmonary arteries and veins. The vessels with the highest degree of stenosis were categorized based on the narrowness of their pulmonary arteries: No stenosis (0%), minimum stenosis (1%−24%), mild stenosis (25%−49%), moderate stenosis (50%−69%), severe stenosis (70%−99%), and occlusive stenosis (100%)(18). In addition, we noted the location of vein stenosis relative to the pericardium and the location of pulmonary artery stenosis relative to the lung.

Next, the degree of bronchus compression was assessed. Moreover, the lung parenchyma, the presence of pleural effusion and atelectasis, calcification of mediastinal lymph nodes, and expansion of the RV and RA were also assessed using additional HRCT measures.

### 2.4 Statistical Analysis

Continuous variables with a normal distribution are presented as mean value ± standard deviation, whereas those with a skewed distribution are reported as median and interquartile range. A *p*<0.05 was considered statistically significant. All statistical analyses were performed using R software (version 4.1.0).

## 3 RESULTS

Table **1** summarises the clinical and radiological features of the 64 FM patients with TB. We performed a detailed analysis of the chest CT imaging features of these patients, and the findings are listed below.

### 3.1 Aberrant Lung Parenchymal Morphology

**Fig. (2):**
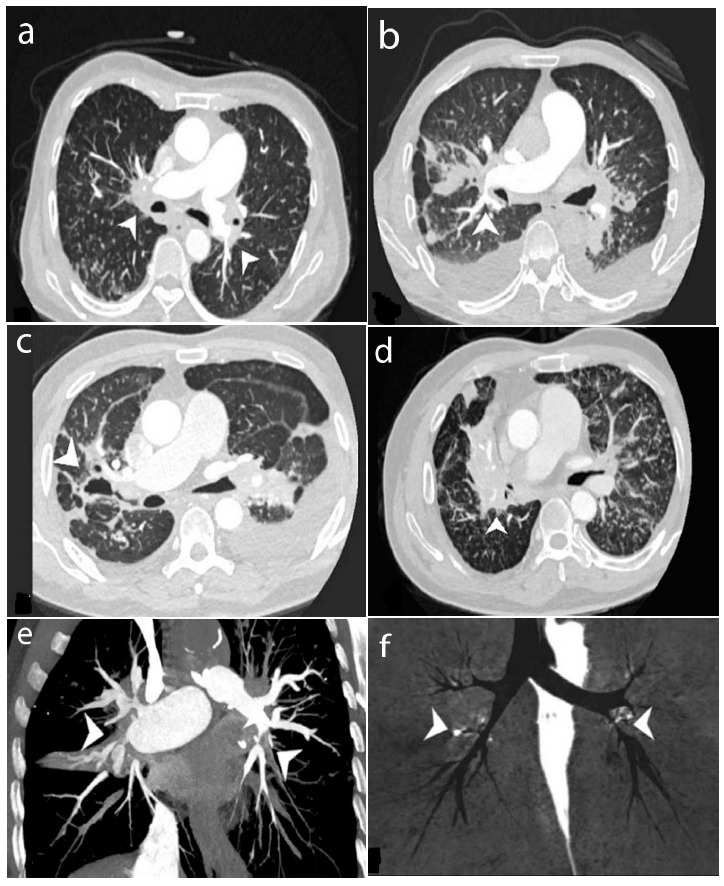
HRCT of TB-FM (different patients). (**a**-**d**) Fibrosis tissue is located at both hilar, causing stenosis of the pulmonary arteries and bronchus (marked by a white arrow). Consolidation (**b**-**c**), cavitation (**c**), pleural effusion (**b**-**c**), and Centrilobular nodules (**d**) are also shown (white arrow). (**e**, **f**) Bronchial and pulmonary arteries in the same segment are prone to stenosis (displayed by a white arrowhead).

Out of the 64 individuals with confirmed TB on HRCT (Fig. **2**), only one patient's TB status could not be determined as active due to a significant pleural effusion. Among the remaining 63 patients, indicators of active TB, such as centrilobular nodules (40, 63%), consolidation (35, 56%), and cavitation (4, 6%), were observed. Additionally, fibronodular scarring was observed in 38 individuals (approximately 60%), predominantly in the upper lobe. These patients also exhibited pleural effusion (17.27%) and emphysema (10.16%).

### 3.2 Mediastinal Alterations

In all 64 individuals, fibroinflammatory reactions were widely dispersed in the mediastinum and bilateral hilar region. During contrast-enhanced imaging, the mediastinal fibrous tissue appeared irregular and poorly delimited, with minimal reinforcement. Enlarged mediastinal lymph nodes were observed in the majority of patients (97%). Among them, 43 patients (67%) had calcified mediastinal and hilar lymph nodes. The maximum short diameters of lymph nodes in the mediastinum ranged from 8.0 mm to 28.0 mm (mean: 16.49±3.85 mm), with most appearing merged and poorly defined.

### 3.3 Cardiac Manifestations

The architecture of the right heart was notably altered, with 43 patients exhibiting right atrial enlargement (67%), 44 showing right ventricular enlargement (69%), and 34 displaying both right atrium and right ventricle enlargement (53%). The CT-measured right ventricular wall thickness ranged from 2.1 mm to 7.4 mm, with a median thickness of 3.6 mm. Further details of these measurements are provided in Table **1**.

### 3.4 Bronchial related Abnormalities

The fibroinflammatory reaction could create segmental and lobar bronchial stenosis as it was disseminated across both hilus. The right middle lobe bronchi were the most commonly affected, with approximately 53% presenting with severe stenosis or occlusion. They were followed by the bronchi of the upper lobes of both lungs, accounting for 16% on the right side and 17% on the left. Meanwhile, the bronchial involvement in the lower lobe of the right lung was uncommon, with around 94% presenting with no or mild stenosis and no severe stenosis and occlusion.

Segmental bronchial stenosis was frequently more severe compared to lobe-level bronchi, with all segmental bronchi exhibiting some degree of severe stenosis or occlusion. The medial and lateral bronchi of the right lung's middle lobe, as well as the apical and anterior bronchi of both upper lobes, showed significant narrowing, with more than 50% of bronchi displaying severe stenosis or occlusion. Particularly, the lateral segment of the right lung's middle lobe (77%) and the apicoposterior segmental bronchus of the upper lobe of the left lung (69%) were the most affected. Approximately 33 patients (52%) experienced partial or complete atelectasis in the middle lobe of the right lung. Table **2** presents the details of the particular bronchial involvement and degree of stenosis.

### 3.5 Pulmonary Artery and Venous Symptoms in 57 TB-related FM Patients

The pulmonary arteries of each lung segment of the upper lobe of both lungs, the middle lobe of the right lung, and the dorsal segment of the lower lobe of the right lung were most seriously affected by fibrotic tissue. The degree of stenosis was mild or moderate, ranging from 42.6% to 57.4%, with the apical and posterior segments of the right lung showing the most pronounced effects. Enclosed by the mediastinal pleura, it was noted that pulmonary artery involvement was predominantly found outside the mediastinal pleura. However, there were a few cases of pulmonary artery involvement within the mediastinal pleura. Specifically, one case involved the anterior pulmonary artery of the upper lobe of the left lung, two cases involved the apical and anterior segments of the right lung, and one case involved the posterior segment and middle lobe of the right lung.

In the intrapericardial region, stenosis of the right upper pulmonary vein was more prevalent (70%), primarily induced by compression from the adjacent dilated right pulmonary artery (n=33, 79%). Conversely, in the extrapericardial segment, stenosis was predominantly observed in the left upper pulmonary vein (79%) and left lower pulmonary vein (83%), attributed to the wrapping of TB-related fibrotic tissue in FM around the hilar region. Furthermore, it was uncommon for the right inferior pulmonary vein to narrow, with 98% of its lumens showing no definite stenosis or only mild stenosis. PA involvement was seen in a total of 57 patients, whereas PV involvement was noted in 82.5% (47 out of 57) of this subgroup. No individuals in this subgroup were found to exhibit PV involvement alone, resulting in vascular stenosis.

**Fig. (3):**
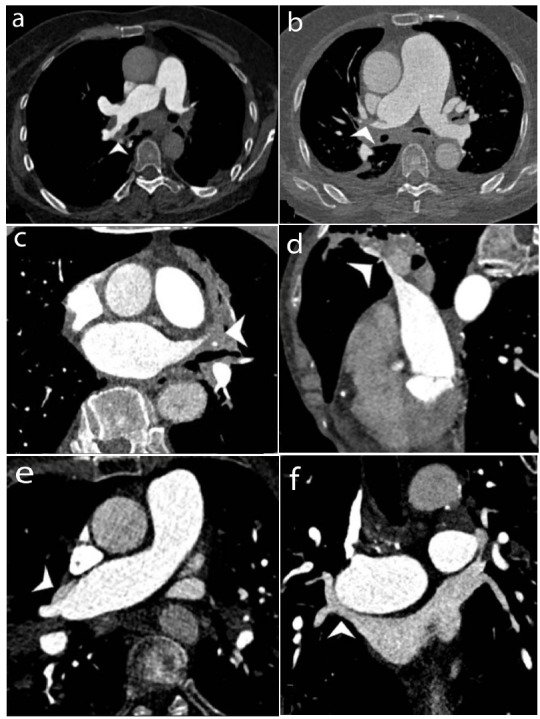
CTPA of TB-FM (different patients). (**a**) The right upper pulmonary artery is wrapped by fibrosis tissues outside the mediastinal pleura (white arrowhead). (**b**) The right upper pulmonary artery occluded inside the mediastinal pleura (white arrowhead). (**c**) The left pulmonary vein occluded inside the pericardial (white arrowhead). (**d**) The right upper pulmonary vein is affected outside the pericardial (white arrowhead). (**e**, **f**) The right upper pulmonary vein is pressed by the dilated right pulmonary artery trunk (white arrowhead).

MPR views offer better imaging insights into the distribution of fibrous tissue. It was found that fibrous tissues were distributed outside the lumens of pulmonary vessels, exerting external pressure and causing vascular stenosis. Additionally, fibrous tissue spread distally along the bronchial sheath from the hilar area. Blood vessel outlines covered in fibrous components often appeared blurred and irregular. Bronchial stenosis and pulmonary artery stenosis in the same segment are frequently observed together (Fig. **3**). The involvement of PA and PV, along with the degree of stenosis, are shown in Table **3**.

## 4 DISCUSSION

FM causes hyperplasia of fibrous tissue, which wraps mediastinal structures and compresses pulmonary arteries and airways, resulting in PH and consequent clinical symptoms [2]. The average age of the patients in this study was 68.5 years, and all were diagnosed with pulmonary TB on chest CT findings. Additionally, 41 patients tested positive for biopsy tissue and sputum acid-fast bacilli staining or mycobacterial culture. Forty patients who underwent right heart cathe-terization were diagnosed with PH (≥20 mmHg), which was consistent with outcomes from previous studies [16, 17].

Unlike other granulomatous infection-related FM [12], 100% of the mediastinal and bilateral hilar structures were involved in the chest HRCT examination of the 64 TB-FM patients, with lesions showing diffuse distribution. This observation aligns with the results of earlier studies where mediastinal and bilateral hilar lesions were present in 85% of 28 TB-FM patients [14]. Therefore, confirming lesions in the mediastinum as fibrous tissue is crucial in the radiologic diagnosis of FM, and a dual-phase CTPA scan can help determine if the lesions are enhanced. Imaging findings of mediastinal lesions, such as diffuse distribution, indistinct borders, calcifications, and no or mild enhancement, may be utilized to distinguish TB-FM patients from other mediastinal disorders (such as tumors) that can lead to extravascular pressure stenosis. Unlike other infections that cause granu-lomatous fibrosing mediastinitis, such as *Histoplasmosis*, TB-FM often involves both hila and rarely affects the superior vena cava, making it a distinguishing point [15]. Another less common form is non-granulomatous fibrosing mediastinitis associated with autoimmune diseases, where fibrotic tissue extensively involves the mediastinum and hilar structures, with calcification being rare. Additionally, retroperitoneal fibrosis, sclerosing cholangitis, and autoimmune pancreatitis are common [13]. Furthermore, in line with findings from other studies, chest CT imaging features of TB-FM include atelectasis, consolidation, emphysema, pleural effusion, pulmo-nary interstitial involvement, mediastinal lymphadenopathy, lymphatic calcification, *etc*.

Some patients may exhibit morphological changes in the right heart. These imaging signs are commonly observed in PH-FM patients and are primarily related to the pathological process of FM [18, 20, 21]. A study by Liu *et al*. also revealed that pulmonary vascular fiber wrapping is a prevalent symptom of FM, and our research provides additional insight into the specific sites affected by TB-FM, extending to the segmental level, including pulmonary arteries, pulmonary veins, and bronchi [14]. The bronchi of the middle lobe of the right lung, along with its segmental bronchi, were shown to be particularly susceptible to fibrous tissue involvement, with over half of the patients experiencing partial or complete atelectasis in the middle lobe of the right lung. This phenomenon is largely attributed to the curved pattern of the bronchus, the placement of the lobe and segmental bronchus bifurcation, and the impact of cardiac beat. In addition, patients with TB-FM often experience recurrent and refractory hypoxemia, as well as other clinical symptoms, as segment-level bronchial stenosis tends to be more severe than lobe-level bronchial stenosis. The presence of bronchial stenosis poses a challenge in FM treatment. While some patients experience a drop in pulmonary artery pressure after interventional treatment to relieve pulmonary artery stenosis, the presence of homosegmental bronchial stenosis causes V/Q imbalance, leading to or aggravating hypooxygenemia.

Dual-phase CTPA scanning was performed in this investigation to provide enhanced visualization of pulmonary artery and pulmonary vein involvement. It was observed that pulmonary artery involvement was shown to be more prevalent than pulmonary vein involvement, aligning with earlier findings [14]. The upper lobe pulmonary arteries and veins of both lungs, the middle pulmonary artery of the right lung, and the dorsal pulmonary artery of the right lung's lower lobe were identified as the most vulnerable to involvement. MPR and careful observation are critical for determining if the major body of the lesion is located inside or outside the pulmonary vascular lumen. Moreover, we discovered that bronchial and pulmonary arteries in the same segment are prone to simultaneous stenosis. This finding holds significance in distinguishing FM from other causes of vascular stenosis. If individuals exhibit severe pulmonary artery involvement without major bronchial abnormalities, the diagnosis of FM becomes questionable. Furthermore, segmental pulmonary artery and vein stenosis are often more severe than lobe-level vascular stenosis due to the higher likelihood of fibrous tissue covering the hilum structures outside the pericardium and beyond the mediastinal pleura. However, there is an exception to the main cause of pulmonary vein stenosis. In most cases, the right upper pulmonary vein lies outside the pericardial cavity owing to anatomical factors, and its stenosis primarily results from compression brought on by the enlargement of the nearby right pulmonary artery trunk as a result of elevated pulmonary artery pressure. Prior to undergoing interventional treatment, such as balloon dilatation and endovascular stents, evaluation of the position of the involved vascular (whether it lies within or outside the pericardium) may assist in predicting potential interventional complications and plan preventative measures accordingly.

Previous research has suggested that because of increased PVR and lower cardiac output in patients with PH, the filling time of pulmonary arteries is prolonged, resulting in more false positives in single-phase CTPA. Guan *et al*. conducted a study comparing single-phase and dual-phase CTPA scans in patients with pulmonary artery embolism. They discovered that dual-phase scans demonstrated better sensitivity, reaching up to 100% [22]. Furthermore, recent research has shown that chest X-rays are useful for identifying and screening PH-FM based on clinical information [23]. By superimposing some typical PH-FM imaging features on clinical data, such as TB history and BNP value, chest X-rays can improve the ability to predict PH-FM, providing new ideas for the comprehensive evaluation of TB-FM patients using imaging techniques. Moreover, CTPA provides a visual representation of the distribution of media-stinal and intrapulmonary lesions, depicting the compression and wrapping of fibrous tissues on adjacent structures. It accurately identifies the responsible blood vessels, thereby guiding the formulation of interventional treatment plans.

In addition to its advantages, this study has several limitations. Firstly, the lack of data on certain clinical indicators resulted in statistical inferences based on a relatively small number of events. Although we excluded most causes of fibrosing mediastinitis other than pulmonary tuberculosis, 23 patients lacked microbiological testing to confirm tuberculosis. The diagnosis of tuberculosis was based on imaging findings, which increases the bias to some extent. Secondly, since this study was designed as a retrospective analysis to identify eligible patients and did not establish a causal relationship or rule out potential lingering confounders, there is a possibility that our study may have been influenced by selection bias. Thirdly, it is important to investigate the differences in radiological characteristics between PH-FM and FM without PH, as well as to explore the correlation between radiological characteristics of PH-FM and the severity of PH, which remain unknown and warrant further investigation in the future.

## CONCLUSION

In conclusion, this study examined the chest CT imaging characteristics of TB-FM using HRCT and conducted an in-depth analysis of the specific pulmonary arteries, pulmonary veins, and bronchi affected by TB-FM down to the segment level. Utilizing dual-phase CTPA scanning, changes in hemodynamic information and the underlying reasons for these symptoms were elucidated. This comprehensive approach can help healthcare practitioners and radiologists evaluate patients thoroughly.

## Figures and Tables

**Appendix 1:** Abbreviations of pulmonary vessels.

PA	Abbreviations	PV	Abbreviations	Bronchus	Abbreviations
right upper lobe pulmonary artery	RUPA	right upper pulmonary vein	RUPV	right upper lobe bronchus	RULB
apical segmental	RUPA -1	apical segmental	RUPV-1	apical segmental	RULB-1
anterior segmental	RUPA -2	anterior segmental	RUPV-2	anterior segmental	RULB-2
posterior segmental	RUPA -3	posterior segmental	RUPV-3	posterior segmental	RULB-3
right middle lobe pulmonary artery	RMPA	right inferior pulmonary vein	RIPV	right middle lobar bronchus	RMLB
right lower lobe pulmonary artery	RLPA	dorsal segmental	RIPV-1	medial segmental	RMLB-1
dorsal segmental	RLPA-1	common basal	RIPV-2	lateral segmental	RMLB-2
common basal	RLPA-2	left upper pulmonary vein	LUPV	right lower lobe bronchus	RLLB
anterior basal	RLPA-3	apical segmental	LUPV-1	dorsal segmental	RLLB-1
lateral basal	RLPA-4	anterior segmental	LUPV-2	anterior basal	RLLB-2
medial basal	RLPA-5	posterior segmental	LUPV-3	lateral basal	RLLB-3
posterior basal	RLPA-6	left lingular vein	LUPV-4	medial basal	RLLB-4
left upper lobe pulmonary artery	LUPA	left inferior pulmonary vein	LIPV	posterior basal	RLLB-5
apicoposterior trunk	LUPA-1	dorsal segmental	LIPV-1	left upper lobe bronchus	LULB
anterior segmental	LUPA-2	common basal	LIPV-2	apicoposterior segmental	LULB-1
lingular segmental	LUPA-3	-	-	anterior segmental	LULB-2
left lower lobe pulmonary artery	LLPA	-	-	lingular bronchus	LULB-3
dorsal segmental	LLPA-1	-	-	left lower lobe bronchus	LLLB
common basal	LLPA-2	-	-	dorsal segmental	LLLB-1
anteromedial	LLPA-3	-	-	anteromedial	LLLB-2
lateral basal	LLPA-4	-	-	lateral basal	LLLB-3
posterior basal	LLPA-5	-	-	posterior basal	LLLB-4

**Note: **PA-pulmonary artery, PV-pulmonary vein.

**Table 1:** Clinical and radiological characteristics of study population.

-	TB-related FM (n=64)
Clinical data	-
Sex ratio, M/F	27/37
Age, median(range), y	68 (50-91)
Smoking	13 (20.31%)
Tuberculosis	-
Certain	18 (28.13%)
Suspected	46 (71.87%)
Cough	41 (64.06%)
Hemoptysis	7 (10.94%)
Repeated pleural effusion	19 (29.69%)
Radiological data	-
Interstitial involvement	3 (0.47%)
Mediastinal involvement	-
bilateral	64 (100%)
unilateral	0 (0%)
Lymphadenopathy	62 (96.9%)
Calcified lymph nodes	43 (67.19%)
RV/RA enlargement	-
RV	43 (67.19%)
RA	44 (68.75%)
Both	34 (53.13%)
RV wall, median(range), mm	3.6 (2.1-7.4)
Pleural effusion	17 (26.56%)
Cavitation (n=63)	4 (6.35%)
Consolidation (n=63)	35 (55.56%)
Centrilobular nodules (n=63)	40 (63.49%)

**Abbreviations: **FM: fibrosing mediastinitis; TB: tuberculosis; RA: right atrium; RV: right ventricle.

**Table 2:** Details of bronchial involvement and degree of stenosis.

Bronchus (n=64)	Degree of Stenosis (%)		
	Non-mild	Middle	Severe-occlusion
LULB	75.00	7.81	17.19
LULB-1	21.88	9.38	68.75
LULB-2	28.13	15.63	56.25
LULB-3	40.63	21.88	37.50
LLLB	65.63	21.88	12.50
LLLB-1	56.25	12.50	31.25
LLLB-2	62.50	12.50	25.00
LLLB-3	56.25	17.19	26.56
LLLB-4	70.31	9.38	20.31
RULB	71.88	12.50	15.63
RULB-1	35.94	10.94	53.13
RULB-2	31.25	17.19	51.56
RULB-3	42.19	15.63	42.19
RMLB	17.19	29.69	53.13
RMLB-1	25.00	15.63	59.38
RMLB-2	15.63	7.81	76.56
RLLB	93.75	6.25	0.00
RLLB-1	50.00	25.00	25.00
RLLB-2	48.44	15.63	35.94
RLLB-3	50.00	12.50	37.50
RLLB-4	68.75	10.94	20.31
RLLB-5	76.56	7.81	15.63

**Note: **See Appendix 1 for abbreviations.

**Table 3:** Details of involvement of pulmonary artery, pulmonary vein and the degree of stenosis.

Pulmonary Blood Vessels (n=57)	Degree of Stenosis (%)	
	Non-mild	Middle-occlusion
-	-	-
PA	-	-
LSPA	-	-
LSPA-1	49.18	50.82
LSPA-2	52.46	47.54
LSPA-3	45.90	54.10
LIPA	-	-
LIPA-1	72.13	27.87
LIPA-2	63.93	36.07
LIPA-3	70.49	29.51
LIPA-4	80.33	19.67
LIPA-5	83.61	16.39
RSPA	-	-
RSPA-1	42.62	57.38
RSPA-2	54.10	45.90
RSPA-3	43.33	56.67
RMPA	57.38	42.62
RIPA	-	-
RIPA-1	47.54	52.46
RIPA-2	62.30	37.70
RIPA-3	75.41	24.59
RIPA-4	80.33	19.67
RIPA-5	100.00	0.00
RIPA-6	96.72	3.28
PV	-	-
LSPV	82.46	17.54
LSPV-1	50.88	49.12
LSPV-2	61.40	38.60
LSPV-3	57.89	42.11
LSPV-4	85.96	14.04
LIPV	85.96	14.04
LIPV-1	80.70	19.30
LIPV-2	92.98	7.02
RSPV	75.44	24.56
RSPV-1	71.93	28.07
RSPV-2	61.40	38.60
RSPV-3	71.93	28.07
RIPV	98.25	1.75
RIPV-1	92.98	7.02
RIPV-2	96.49	3.51

**Note: **See Appendix 1 for abbreviations.

## Data Availability

The data and supportive information are available within the article.

## LIST OF ABBREVIATIONS

CT: Computed tomography
CTPA: Computed tomography pulmonary angiography
FM: Fibrosing mediastinitis
HRCT: High-resolution computed tomography
MPR: Multiplanar reformation
PAWP: Pulmonary artery wedge pressure
PH: Pulmonary hypertension
PVR: Pulmonary vascular resistance
RAA: Right atrial area
RAP: Right atrial pressure
RHC: Right heart catheter
TB: Tuberculosis
HRCT: High-resolution CT
SPAP: Systolic pulmonary artery pressure
mPAP: Mean pulmonary arterial pressure

## STANDARDS OF REPORTING

STROBE guidelines were followed.
